# Simulation- and Metamodel-Based Multi-Objective Optimization for Sustainable Building Retrofit Across Climatic Conditions

**DOI:** 10.3390/ma19081649

**Published:** 2026-04-20

**Authors:** Sk. Reza-E-Rabbi, Muhammed A. Bhuiyan, Guomin Zhang, Shanuka Dodampegama, Kanishka Atapattu

**Affiliations:** 1 Civil and Infrastructure Engineering Department, School of Engineering, RMIT University, Melbourne 3001, Australia; s3990455@student.rmit.edu.au (S.R.-E.-R.); s3975925@student.rmit.edu.au (S.D.); kanishka.atapattu@rmit.edu.au (K.A.); 2 Centre for Future Construction, RMIT University, Melbourne 3001, Australia

**Keywords:** building retrofit, multi-objective optimization, optimization framework, climatic conditions, sustainable buildings

## Abstract

Building retrofit optimization has gained increasing attention as a pathway to improve energy performance and support sustainability. This review examines 162 studies and synthesizes simulation-based (SBMOO) and metamodel-based (MBMOO) multi-objective optimization techniques for building retrofit across climatic conditions. The review also analyzes passive, active, and combined retrofit strategies and evaluates how climatic context influences their suitability and performance. Passive strategies typically involve envelope- or material-related upgrades, whereas active strategies focus on building systems. Energy efficiency, comfort, cost-effectiveness, and environmental impact are identified as the major performance metrics for retrofit evaluation. Sustainability metric such as life cycle assessment (LCA) has yet to be used adequately to evaluate retrofit measures, while social objectives are also less explored. SBMOO provides robust optimization but can be computationally intensive, whereas MBMOO improves computational efficiency through surrogate modeling but depends strongly on dataset quality, sampling strategy, and surrogate model selection. In contrast to earlier reviews that usually emphasize either optimization techniques or retrofit measures independently, this study integrates optimization pathway comparison with climate-based analysis of retrofit strategies. The review also finds that most studies are highly case-specific, limiting transferability across climates, building types, and retrofit contexts. Therefore, this work proposes a synthesized framework to support structured selection of baseline modeling and optimization pathways for future retrofit studies. Overall, the review identifies current methodological trends, key research gaps, and future directions for more consistent and climate responsive retrofit decision-making.

## 1. Introduction

The building sector is crucial in environmental sustainability, accounting for approximately 40% of global energy-related carbon emissions due to its high energy consumption [1]. Retrofitting existing buildings offers a critical pathway to achieve carbon neutrality, as existing buildings vastly outnumber newly constructed ones. To achieve net-zero emissions by 2050, 20% of the existing building stock, as well as all new buildings, must meet energy efficiency standards [2]. Therefore, retrofitting is a key strategy for achieving sustainable buildings by improving energy efficiency, reducing CO_2_ emissions and energy costs, and enhancing indoor comfort [3].

Retrofitting strategies can be classified under two main approaches, passive and active, which vary in cost, effectiveness, and practical feasibility. This distinction helps in selecting appropriate measures based on building conditions and retrofit goals [4]. The passive approach improves a building’s energy efficiency by optimizing its structure, material selection and design, such as enhancing insulation or glazing [5,6]. In contrast, the active approach involves upgrading or replacing energy-consuming systems, such as HVAC units and lighting [7]. Additionally, combining passive and active retrofitting strategies can significantly boost a building’s thermal comfort and energy efficiency [8]. Moreover, integrating renewable energy sources into this hybrid strategy can enhance sustainability, offering long-term economic and environmental advantages [2]. Retrofit performance is typically assessed using multiple criteria, including reduction in energy consumption, maximization of thermal comfort, cost efficiency, and minimal environmental impact. The effectiveness of various retrofit strategies is analyzed based on these most desired outcomes [9,10].

Climate conditions significantly affect these building retrofit results, as heating and cooling demands, solar gains, and thermal comfort responses differ across hot, humid, dry, and cold environments [11]. These conditions are typically represented through weather files that drive energy performance calculations. In retrofit projects, the typical weather data extracted from the historical climate pattern is ubiquitous for estimating energy savings [12]. Each climatic condition has a unique demand on building performance. Therefore, the retrofit strategies should vary in different climatic situations [13]. The effectiveness of retrofit strategies may also vary by orientation and treatment conditions, as these factors can affect the performance and durability of building components [14,15]. Since retrofit decisions balance multiple conflicting objectives such as energy use, cost, thermal comfort and environmental impact, multi-objective optimization (MOO) has become an important decision support approach [16]. MOO approaches can be broadly categorized into simulation-based multi-objective optimization (SBMOO) and metamodel-based multi-objective optimization (MBMOO). In SBMOO, the optimization tool works with a simulation engine, utilizing its evaluations to find optimal solutions. Conversely, MBMOO relies on a surrogate model or metamodel that approximates the behavior of the simulation engine. Before it can be used for predictions, the surrogate model must be trained and validated on an extensive dataset to ensure accuracy [17].

While several review papers have examined building retrofits and optimization from different perspectives, important gaps remain in the existing literature. Previous studies analyzed different passive and active retrofit measures under climate change, but within a specific regional context and without linking these measures to retrofit optimization pathways. These works have provided an overview of methodological aspects, measures, and decision-making tools. However, the role of climatic conditions in selecting retrofit measures and a comparative assessment of simulation and metamodel-based MOO pathways have not been comprehensively addressed. Earlier works often addressed retrofit measures in separate categories, lacking a comprehensive synthesis of passive, active, and combined strategies, along with the associated trade-offs across various performance objectives. Moreover, limited attention has been given to the transferability of optimization outcomes, particularly regarding case-specific baseline modeling and surrogate model selection in MBMOO applications [18,19,20,21,22,23].

The above limitations also indicate the need to establish a clearer basis for climate responsive retrofit optimization and decision-making. Therefore, our work also provides a novel framework for retrofit optimization as a structured decision support pathway derived from recurring patterns and limitations identified in the literature. This workflow is also aligned with real world retrofit practices, where interventions are often implemented through a limited number of measures. The proposed framework aims to connect retrofit measure selection, climatic conditions, performance metrics and optimization pathways for more effective retrofit planning.

Accordingly, the objectives of this review are: (1) to critically examine active, passive and combined retrofit strategies along with their trade-offs across major performance metrics; (2) to evaluate the impact of climatic conditions in selecting retrofit measures; (3) to compare SBMOO and MBMOO pathways in terms of workflow, applicability, strength and limitations; (4) to develop a structured framework for practical and transferable retrofit decision-making.

The remainder of this paper is organized as follows. Section 2 describes the review methodology and emerging research trends. Section 3 critically examines retrofit measures and their selection strategies across climatic conditions, while Section 4 delves into specific performance indicators relevant to building retrofits. Section 5 systematically examines and compares simulation-based and metamodel-based optimization approaches, including algorithms, tools, platforms, and metamodels. Based on the reviewed evidence, Section 6 presents a decision support framework for retrofit optimization. Section 7 concludes the review with key findings and future research directions.

## 2. Review Methodology

A systematic methodology was employed to identify major trends and key themes in energy optimization for building retrofitting within the selected literature base. In this approach, identifying relevant research articles was crucial to ensure the review accurately captured the state of the art in this rapidly evolving field. After selecting the relevant literature, a bibliometric analysis was conducted. This analysis examined keyword co-occurrence, highlighting significant themes and trends within the literature, enabling a clear view of how these research areas have evolved, and providing insights into the dominant trends and emerging topics in building retrofitting and energy optimization.

### 2.1. Data Collection and Search Strategy

The research began with a preliminary literature review to broadly map the energy optimization domain in building retrofitting. This initial review helped identify four critical areas essential for understanding the field, as summarized in Table 1. A dedicated search string was developed for each identified area to capture the relevant literature comprehensively. Moreover, including prediction related keywords in the search strings ensured that MBMOO studies leveraging machine learning models were comprehensively captured. Furthermore, overlapping terms in the string were intentional to ensure inclusivity across different phrasing styles used in the literature. This approach helped to capture a broad range of passive, active, and combined strategies, as well as optimization approaches and prediction techniques applied across various retrofit contexts.

Scopus was selected as the primary database for this search due to its extensive collection of research papers in construction engineering and related disciplines. Moreover, many studies indexed in other databases (e.g., WOS) are also included in Scopus, making it a reliable and comprehensive source for capturing relevant literature [24]. The database was considered to provide a broad and consistent literature base for the review and the findings are interpreted within its context. To ensure focused and relevant results, the search field in Scopus was limited to “Article title, Abstract, Keywords.” The search covered the period from 1 January 2015 to 30 December 2025, to capture the most recent decade of research in this rapidly evolving domain. This filter was implemented to maintain the relevance of the review dataset, while some secondary relevant articles might be excluded. Only documents written in English and categorized as articles, conference papers, conference reviews, reviews, or book chapters were considered for inclusion in the review.

A comprehensive search string was initially constructed by combining all four search strings using the “AND” command. However, this approach returned only eight relevant papers, which were insufficient for a robust analysis (Table 2). To expand the search and ensure a broader scope, the search strategy was refined by using various combinations of three search strings, ensuring that the first search string (focused on types of retrofit measures) was consistently included in each combination. This revised approach substantially increased the number of relevant articles, ultimately resulting in the selection of 162 papers from the Scopus database for a more thorough review (Table 2). A set of inclusion and exclusion criteria was also applied to ensure the selected literature’s relevance and quality. These criteria are summarized in Table 3.

Using the search combinations along with the inclusion and exclusion criteria, those 162 papers were screened step by step. These papers were subsequently subjected to a detailed bibliometric analysis, which helped identify key themes and emerging trends within the selected literature on building energy optimization. This body of literature also served as the foundation for the subsequent review sections, where representative studies are discussed in detail to examine retrofit measures, climatic conditions, performance metrics and the application of optimization approaches, focusing on SBMOO and MBMOO. These insights gave a comprehensive understanding of the current research landscape and the strategies employed to enhance building efficiency.

### 2.2. Bibliometric Analysis

To identify prevailing themes and trends in energy optimization for building retrofitting, a bibliometric analysis was performed using VOSviewer (1.6.19), an open-source software designed for constructing and visualizing bibliometric networks, such as keyword co-occurrence networks. VOSviewer excels in identifying research clusters, mapping emerging trends, and analyzing the structure of research fields [25]. By visualizing connections between keywords and research topics, this tool helps researchers understand the relationships and developments within a particular domain, providing insights into the most active and impactful areas.

The authors concentrated on keyword co-occurrence in this analysis to reveal the principal research clusters within the selected papers. Before the analysis, a data cleaning process was conducted to enhance accuracy. This involved merging similar keywords to prevent redundancy and removing irrelevant keywords, such as “article” and “country,” to maintain the focus on pertinent research terms. Initially, 1253 keywords were identified across the relevant articles. To refine the analysis, the authors narrowed it down to 32 keywords by setting a threshold, requiring each keyword to appear at least four times. The clustering resolution was set to 2 to identify research clusters effectively, and the minimum cluster size was adjusted to 8. These parameters were chosen to allow the merging of smaller clusters, ensuring a more coherent and insightful data analysis.

**Figure 1:**
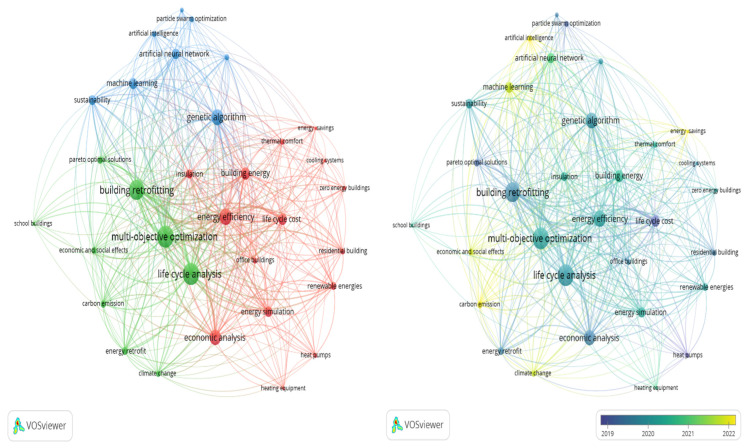
Network of keyword co-occurrence and time overlay visualization.

Figure 1 illustrates the keyword co-occurrence network and a time overlay visualization. The left portion of Figure 1 highlights three main clusters. In this network, the size of the circular nodes represents the frequency of keyword occurrences, indicating the strength of their presence in the literature. The width of the lines connecting the nodes reflects the strength of co-occurrence between two keywords, signifying how often they appear together in the same articles. This network provides a clear representation of the interconnections between research topics to identify central themes and collaborative relationships within the field of building energy optimization and retrofitting [26]. The green cluster, in the left portion of Figure 1, is associated with building retrofitting, multi-objective optimization, and life cycle analysis (LCA), representing the core concepts and methodologies used in optimizing building energy performance. This cluster indicates a strong focus on integrating multiple objectives and assessing the life cycle impacts of retrofitting measures. The red cluster, which includes keywords such as energy efficiency, life cycle cost (LCC), economic analysis, and energy simulation, reflects research centered on energy optimization’s economic and simulation aspects. This cluster emphasizes evaluating retrofitting strategies’ financial implications and energy performance through simulation-based approaches. Meanwhile, the blue cluster, containing nodes such as artificial intelligence (AI), machine learning (ML), genetic algorithms, and sustainability, represents the advanced computational techniques and their application to sustainable building practices. This cluster highlights the growing interest in leveraging AI and ML to enhance the efficiency and sustainability of retrofitting measures.

Furthermore, a time overlay visualization was performed to identify research trends over time, as shown in the right portion of Figure 1. In this visualization, more recent research trends are represented in yellow, while older trends are shown in purple. The yellow nodes indicate a recent surge in interest in AI, ML, carbon emissions, and energy-saving measures’ economic and social effects. This trend suggests a shift towards integrating advanced computational techniques focusing on reducing carbon footprints and assessing the broader impacts of retrofitting. While keywords related to multi-objective optimization, LCA, and energy simulation appear as older trends, they remain integral to the current research landscape. This continuity implies that while these concepts have been established for some time, they continue to be relevant and are now being integrated with newer trends, such as AI and ML, to enhance the effectiveness and sustainability of energy optimization in building retrofitting.

## 3. Review of Retrofit Measures

The bibliometric analysis in the previous section has underpinned the importance of building retrofitting by having a larger node. Therefore, this section highlights different retrofit measures implemented in the literature and analyzed them critically. Moreover, climate conditions are an essential component of sustainable building design, and this section also examines how climate conditions influence the selection of retrofit strategies. It further indicates that the suitability of key material-based retrofit measures varies across different climates.

### 3.1. Retrofit Approaches: Passive, Active, and Combined

Passive strategies (PS) generally emphasize the augmentation of the building envelope (e.g., insulation, window enhancements, shading) to improve thermal resistance and diminish reliance on active heating or cooling systems. On the contrary, active strategies (AS) encompass enhancements to HVAC systems, lighting, and additional infrastructure, often requiring energy inputs for operation.

Implementing passive measures, such as applying sprayed polyurethane and expanded polystyrene insulation on walls and rooftops, installing double-glazed windows, adding overhangs, and introducing rooftop shading, substantially improves thermal performance and energy efficiency [27,28]. Active measures, such as HVAC upgrades and advanced lighting systems, also reduce energy usage significantly but rely more heavily on regular maintenance and user engagement (e.g., lighting preferences) [29]. Additionally, studies [30] suggest that daylight sensors, lighting control systems, and energy-efficient lighting can reduce lighting energy consumption by up to 90% compared to conventional systems. PS frequently have longer payback durations due to their substantial upfront expenditures, yet they generate savings over time by reducing heating and cooling demands. Conversely, AS, such as the implementation of LED lighting, offer shorter payback periods and are regarded as economically viable [31]. Moreover, strategies like roof insulation can have a significant effect on reducing life cycle costs (LCC) and enhancing thermal comfort [32]. Furthermore, overhangs and green roofs lower energy demand and reduce greenhouse gas emissions. In contrast, AS often involves higher operational energy demand, yet may achieve emissions mitigation when integrated with optimized systems [33].

**Figure 2:**
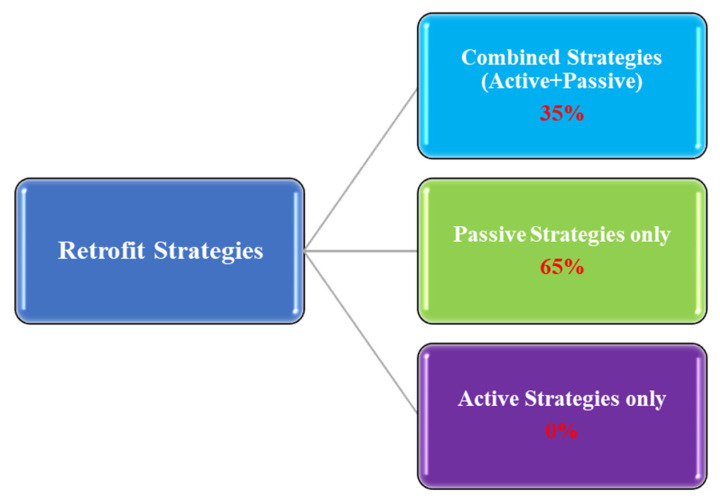
Retrofit strategy preferences: insights from analyzed studies.

It has been observed that most analyzed studies have focused solely on PS (Figure 2). Passive retrofit options such as glazing, insulation, shading, etc., are easy to model within the energy simulation workflow and involve less operational uncertainties than active systems. Their performance is directly linked to climatic conditions, which also helps to do the comparative analysis across regions. Moreover, due to their long-term influence on cooling and heating load demand, PS is commonly adopted in residential building retrofits. These factors may have contributed to their stronger presence in retrofit analysis [9,34]. However, using only passive measures has certain drawbacks. PS inherently relies on environmental conditions and can only offer limited control over indoor temperatures. Therefore, it might not be sufficient to maintain occupant comfort during extreme weather events. Effective passive retrofits, such as enhanced insulation, sophisticated glazing, or thermal mass, often require substantial investment in design and materials. However, existing retrofitting structures can be particularly complex and expensive if the building was not designed with passive techniques in mind. Additionally, after a certain point, further investments in insulation or glazing provide only marginal energy savings [35,36,37].

On the other hand, relying on AS only can lead to higher operational costs as active systems require energy to operate. Additionally, solely utilizing active cooling, heating, or lighting measures will lead to higher energy consumption and a larger carbon footprint [38]. Moreover, active systems such as HVAC are essential for thermal comfort; however, their performance may be suboptimal without optimizing PS. Due to these factors, in the analyzed studies, AS have not been considered alone, as their effectiveness depends significantly on the building’s underlying passive characteristics (Figure 2). Therefore, authors often integrated PS when implementing AS to achieve better outcomes [39].

A combined retrofit strategy capitalizes on the strengths of both passive and active techniques, leading to enhanced energy efficiency, cost savings, reduced environmental impact, and improved overall building performance [8,40,41]. The building may not achieve optimal energy savings without combining passive and active measures. For instance, depending on passive insulation without implementing effective heating and cooling systems may yield discomfort during extreme conditions. Conversely, reliance on HVAC systems without improved insulation may lead to excessive energy usage. Studies demonstrate that combining insulation (walls, roof, floor, windows) with mechanical ventilation and boilers can cut energy costs, demand, and carbon emissions by nearly 50% and reduce investment costs by 60% [42,43,44]. Also, these types of approaches significantly improved the energy performance of non-residential buildings, reducing energy consumption and thermal demand while enhancing thermal comfort [45,46].

The literature indicates that approximately one-third of the studies have focused on combining active and passive strategies for retrofitting buildings (Figure 2). Despite the promising potential of combining active and passive strategies, limited research has been conducted in this area, indicating significant scope for further exploration. However, combined retrofit strategies are expensive and intricate, requiring substantial initial investments, prolonged payback durations, and potential operational disturbances [47]. Additional challenges arise from structural constraints, climatic fluctuations, and continuous maintenance requirements. Regulatory constraints and occupant behavior may also affect their effectiveness and economic sustainability [48].

Furthermore, in the case of retrofitting buildings, the goal of zero or low-carbon energy can be achieved by including renewable energy measures, which will eventually offset the energy consumption. Therefore, it has been observed from the analyzed literature that incorporating combined retrofit strategies also includes renewable energy (RE) as retrofit measures. However, only a few studies paired passive measures with RE as a retrofit approach. RE technologies such as solar PV, heat pumps, and wind turbines are commonly used in retrofitting [10]. The electricity supply from a PV solar panel has been illustrated as an energy-saving measure with minimal environmental impact. Scholars depicted that its integration with efficient envelopes decreased primary energy consumption by 71.35% and CO_2_ emissions by 73.42% [49,50]. However, the high upfront costs associated with PV installation and the challenges posed by elevated outdoor temperatures are major barriers to the broader adoption of these renewable energy systems [7,51].

Overall, the reviewed literature indicated that PS remains the favorable choice in retrofit analysis. In contrast, the combined implementation of PS and AS tends to provide benefits when comfort, cost, and emissions are considered together.

### 3.2. Choice of Retrofit Measures in Different Climatic Conditions

**Figure 3:**
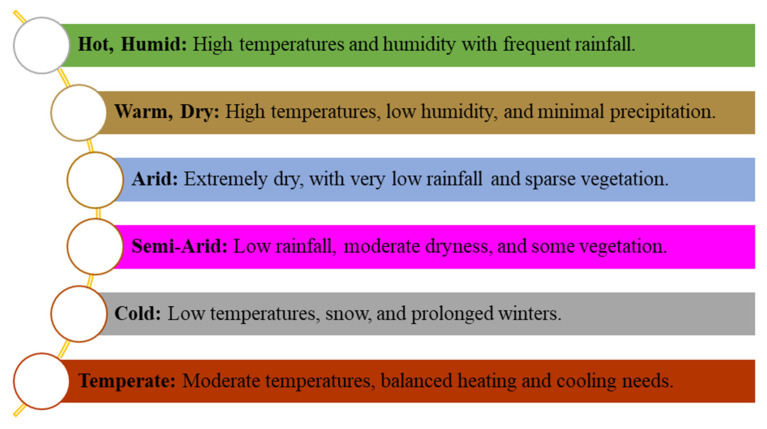
Different climate conditions.

To formulate effective retrofit measures that enhance energy efficiency, it is essential to understand the specific climatic conditions and regional requirements [52]. Climate variability, particularly shifts in external thermal stresses, can significantly influence the efficiency of heating and cooling systems, necessitating adjustments in strategies to suit the local environment [53]. However, the climate conditions for different regions have been shown in Figure 3.

Our climatic classifications are not strictly based on the Köppen–Geiger system but exhibit a general correspondence with it. They are not intended to serve as strict meteorological groups. They have been organized to accurately represent how retrofit studies usually describe and compare climate conditions. Therefore, the groups are organized according to thermal performance and the applicability of retrofitting, which is a prevalent methodology in the field of building energy research [7]. The thermo-radiative environment is pivotal in determining cooling and heating loads, especially in hot and humid climates, where factors such as air infiltration and vegetation humidification become particularly relevant [33,54]. Researchers [55] have highlighted several effective passive cooling strategies (PCS) universally applicable across hot climates, including optimizing exterior wall properties, window glazing, and natural ventilation systems.

Although the window-to-wall ratio is not a typical retrofit option, it emerges as a key PCS in hot and humid conditions, whereas roof properties hold greater importance in hot, dry climates [56]. In hot, humid conditions, limited airflow, and hot, dry conditions, extreme temperature fluctuations could challenge implementing these strategies. Tropical climates generally align with the hot and humid category. External insulation is essential to maintain thermal performance for buildings in tropical and arid regions [57]. In contrast, internal insulation more effectively regulates indoor temperatures in temperate and cold climates [56,58]. In tropical and arid areas, the high cost of external insulation can be an issue, while in temperate and cold climates, moisture problems may arise with internal insulation [59]. Consequently, active solutions like lighting and air conditioning enhancements offered better cost-effectiveness for retrofitting endeavors in hot and humid environments [60].

In a sub-arctic (dark and cold) climate zone, various types of insulation, such as polyisocyanurate, vacuum-insulated panels, expanded polystyrene insulation, mineral wool, and cellulose, can be selected as retrofit measures for roof and wall insulation. For windows, double or triple glazing should be the preferred choice. Moreover, improving heat recovery ventilation could be an effective choice for active retrofitting [41]. Glazing systems and thermal insulation also provide excellent opportunities to enhance the 3E (energy, economy, and environment) in cold climates. Combining several passive approaches, such as PCM, thermal insulation, reflective paint, shading, and glazing, can also offer the best opportunities to improve the 3E in hot conditions [61]. The overhang specification could be selected as passive strategy to enhance the energy performance of buildings across different climatic regions, such as cold, mild, warm-dry, and warm-humid [62].

In contrast, lighting, heating/cooling systems, and water heating systems are depicted as the preferred AS in cold areas [63,64]. However, the effectiveness of insulation can be reduced by extreme cold, while glazing and reflective paints may degrade due to intense dust and heat. Again, there is a risk of condensation and mold in warm-humid areas, which may affect the performance of passive and active measures like shading and air conditioning. Mediterranean climates typically have characteristics of warm and dry climate. In the Mediterranean climate, AS include heat generators and heating/cooling systems, while PS consist of insulation and window types. However, temperature fluctuations in Mediterranean climates may hinder the efficiency of heating and cooling systems [46,65]. In addition, solar PV panels are implemented as RE solutions in hot, humid, warm, and cold regions. Nevertheless, high humidity can diminish the efficiency of PV in hot and humid region, while limited sunlight and snow limit the PV performance in cold climate [50,51,64].

Furthermore, another retrofit strategy such as integrating phase change materials (PCM) in building envelopes can offer varied benefits depending on the climate. In tropical regions, PCM integration may yield little advantages due to the continuous high temperatures. However, in arid climates, applying PCM to the outer surface of a building is beneficial, helping to regulate temperatures by absorbing excess heat during the day and releasing it at night. For temperate and cold climates, embedding PCM within the central or inner layers of the building envelope leads to more efficient thermal management, reducing heating requirements [5]. The benefit of integrating PCM in a tropical region can be limited due to high constant temperatures, whereas arid climates may encounter difficulties due to high heat loads, possibly surpassing PCM’s capabilities.

Generally, the above-discussed studies highlighted that retrofit measures are strongly climate dependent. Envelope-related passive measures are the dominant choice in hot and cold climates. The combined application of AS and PS becomes significant when ventilation, cooling and system efficiency play an important role. This demonstrates the importance of climate-based choice of retrofit measures and further integrates this part into the optimization process for better trade-offs among energy, cost, comfort and environmental impact. It should also be noted that most of these studies rely on weather files, as they usually provide a consistent and practical basis for retrofit analysis. However, future climatic conditions may also need to be considered, as retrofit performance can vary under changing climatic conditions. Moreover, Table 4 summarizes the common retrofit strategies for various climate conditions and the strength of supporting evidence from the reviewed studies.

## 4. Performance Metrics in Building Retrofits

In the context of a retrofit project, the effectiveness of a design can be evaluated using specific performance indicators. These indicators help determine the most suitable retrofit strategies tailored to achieve objectives. The key performance indicators are energy consumption reduction, cost efficiency, improved comfort, and minimized environmental impact. From a holistic perspective, cost efficiency and thermal comfort are economic and social objectives, while energy demand and consumption fall under environmental objectives [18].

**Figure 4:**
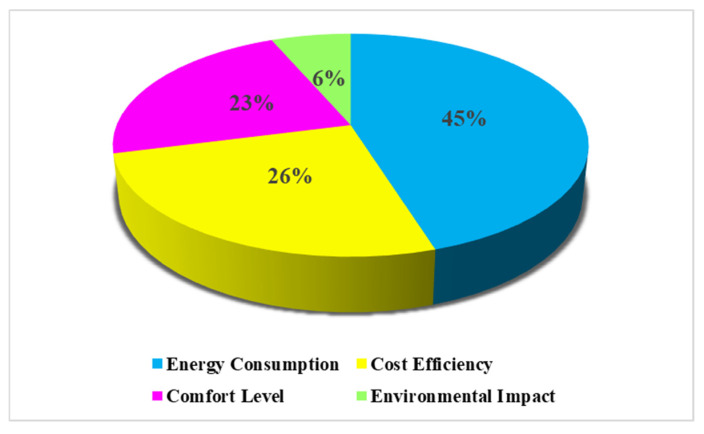
Distribution of focus in retrofit objectives.

**Figure 5:**
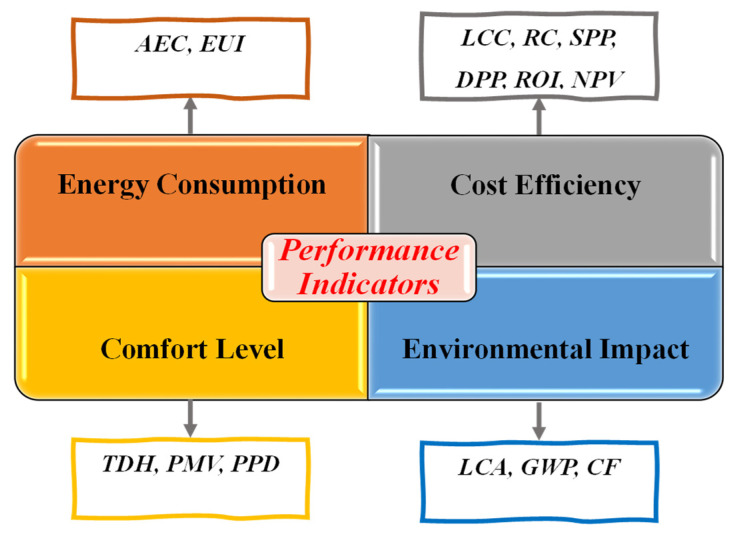
Performance indicators.

Based on the analyzed literature, Figure 4 exhibits the focus of these four principal objectives in building retrofit optimization. It emphasizes the prioritization of energy consumption (45%) and cost efficiency (26%) as objectives over environmental impact (6%) and comfort level (23%). Environmental and social objectives received limited attention due to their complex evaluation procedure compared to energy and cost. The energy usage and retrofit cost results are obtained from the simulation outputs and cost calculations, while social and environmental metrics require additional datasets, post-processing and assumptions. The data collection related to occupant behavior and the incorporation of user dependent indicators into the optimization has proven to be challenging [76,77]. Additionally, life cycle assessment (LCA) is an important performance indicator related to environmental objectives and can evaluate long term environmental trade-offs. However, the difficulties of collecting embodied impact data, service life assumption and the complexity of integrating life cycle calculation into the SBMOO process pose challenges to its wider application [78,79]. Therefore, retrofit analysis remained more focused on energy and financial gains, whereas environmental and social performance indicators are insufficiently considered [56,63,80]. Moreover, some specific metrics are commonly used to evaluate how effectively these objectives are achieved (Figure 5).

The reduction in annual energy consumption (AEC) is typically evaluated through key metrics like energy use intensity (EUI) and annual energy usage (kWh/m^2^), both of which are used to measure the energy efficiency of a building. EUI is considered more comprehensive because it accounts for all forms of energy consumption, including electricity, cooling, and heating. However, EUI might fail to present variations as it is data-intensive, while AEC has limitations in identifying specific inefficiencies as it lacks granularity [81,82]. When it comes to thermal comfort, the evaluation is often tied to metrics like thermal discomfort hours (TDH), predicted mean vote (PMV), and predicted percentage of dissatisfaction (PPD). TDH is a straightforward indicator that reflects how many hours the indoor temperature exceeds comfort thresholds in a year. On the other hand, PMV and PPD are advanced measures that factor in environmental variables such as temperature, humidity, airflow, occupants’ activity level, and clothing insulation. Additionally, PPD quantifies the proportion of people who are not satisfied with the thermal environment, while PMV measures the overall comfort level. Nevertheless, TDH provides a simple measure but is insensitive to variables like humidity, airflow, or occupant behavior. PMV and PPD offer a broader analysis, but they may not provide actual conditions consistently, as they depend on assumptions regarding activity levels, clothing, and environmental factors [83,84].

Cost efficiency is evaluated using metrics like life cycle cost (LCC), retrofit cost (RC), net present value (NPV), return on investment (ROI), simple payback period (SPP), and discounted payback period (DPP). LCC represents all costs of a retrofit option throughout its lifespan, while NPV assesses the net value of inflows and outflows, with a positive value indicating profitability. In addition, ROI quantitatively measures the financial gains over time. Moreover, SPP shows the time to recover the investment, and DPP refines this by accounting for discounted cash flows [61,85]. LCC provides an overview of comprehensive costs, but it needs extensive data. Additionally, ROI and NPV can illustrate profitability but rely on accurate financial projections. Furthermore, SPP and DPP are useful for quick evaluation but may inadequately address long-term implications.

The mitigation of environmental impact is mainly related to emissions. In this scenario, LCA, global warming potential (GWP), and carbon footprint (CF) are key in evaluating building performance. LCA is utilized systematically to evaluate the environmental performance of products throughout their life cycle. It is frequently regarded as a comprehensive “cradle to grave” methodology for assessing environmental impacts, but it might be complex as it is data-intensive. However, CF is a specific performance indicator that quantifies the aggregate GHG emissions from building operations. GWP is an important metric that assesses the influence of various gases on global warming. Although CF and GWP provide specific insights, they are limited to emissions, neglecting broader effects [86,87]. Additionally, embodied carbon is necessary to consider in retrofit evaluation along with the operational emissions. Retrofit strategies such as glazing, insulation, and PCM involve upfront emissions from material production and installation. Therefore, considering both embodied and operational emissions can provide environmental benefits from selected retrofit measures over the building life cycle [73,88,89].

Practically, the above discussed performance metrics are conflicting with each other, and they do not perform independently. For instance, some measures that reduce the LCC may minimize the life cycle environmental impact. In contrast, strategies that help to improve the comfort level can increase the retrofit cost. Similarly, low-cost retrofit measures may be useful but may provide weaker long term environmental benefit than solutions with expensive initial investment. Therefore, these interactions highlight the significance of multi-objective retrofit optimization where suitable solutions are identified by balancing these conflicting objectives [56,63,80].

## 5. SBMOO vs. MBMOO in Building Retrofits

The retrofit strategies discussed in Section 3 and the performance indicators summarized in Section 4 establish the basis for multi-objective retrofit optimization. Therefore, this section reviews the optimization pathways used to identify balanced retrofit solutions. Optimization involves searching for solutions by considering all the constraints while intending to maximize or minimize one or more objectives. However, in some cases, decision-makers want to establish a trade-off between two or more objectives that can be optimized consecutively. Generally, simultaneous optimization of these conflicting objectives, such as maximizing the level of thermal comfort while minimizing the cost of retrofit measures, is often referred to as a multi-objective optimization (MOO) problem. After all, in an MOO problem, a set of solutions is typically obtained rather than a single solution. The optimization of multiple conflicting objectives can be achieved by following two different approaches, the simulation-based (SBMOO) and the metamodel-based multi-objective optimization (MBMOO) approaches. Nevertheless, in the mid-1980s, a multi-objective genetic algorithm (GA) was introduced [90]. The existing literature indicates that genetic algorithms (GAs) are widely regarded as the most potent and robust heuristic method for addressing MOO challenges within the area of building optimization [91,92,93,94,95]. The following section outlines key GA concepts and commonly used variants, then compares SBMOO and MBMOO workflows, and finally, summarizes the tools, platforms, and metamodels adopted in the reviewed literature.

### 5.1. Key Concepts and Parameters of GAs in MOO Processes

**Figure 6:**
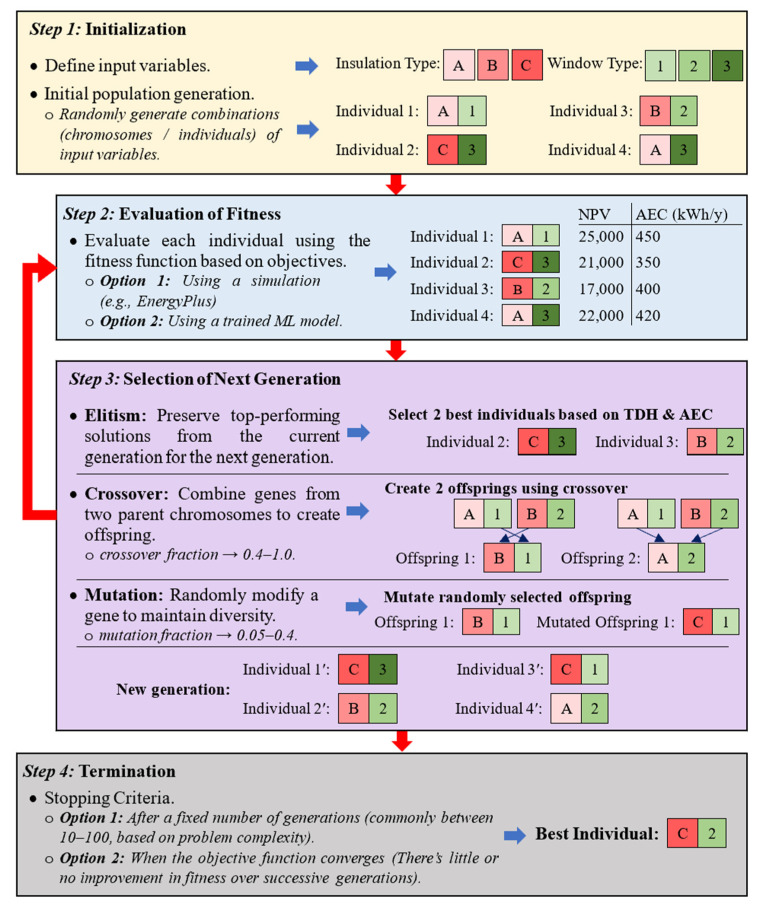
GA process illustrated with a building retrofit example.

GAs are widely used in building retrofit optimization due to their ability to handle conflicting objectives and nonlinear search spaces. The key concepts of GAs in MOO have been analyzed using a building retrofit example for easy understanding (Figure 6). At the end of the procedure discussed in Figure 6, a collection of potential alternative outcomes is achieved. In the post-optimization phase, researchers frequently apply Pareto-based optimization or multi-criteria decision-making (MCDM) techniques to find a set of best trade-off solutions [96,97,98,99,100].

### 5.2. Types of GAs in Building Retrofitting

**Figure 7:**
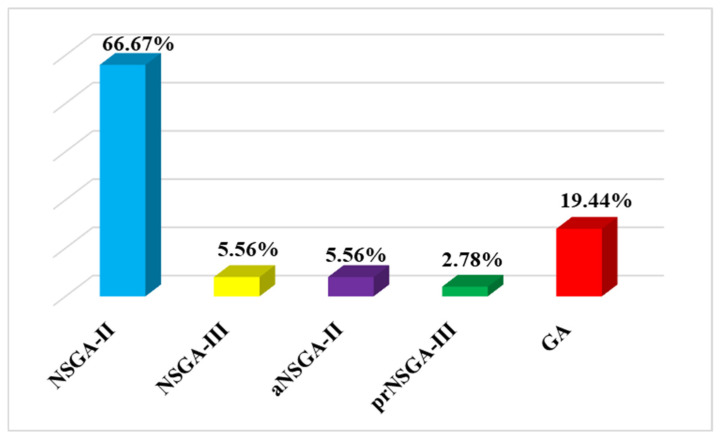
Implementation of different GAs among the analyzed studies.

There are some commonly implemented GAs for solving MOO problems in the context of building retrofitting. The Non-dominated Sorting Genetic Algorithm (NSGA-II) is widely implemented and recognized as an effective multi-objective evolutionary algorithm (MOEA) for optimizing building retrofits (Figure 7). This addresses challenges that traditional gradient-based techniques face in dealing with nonlinear and complex interactions. NSGA-II applies a non-dominated sorting procedure to evaluate population fitness, facilitating convergence to the true Pareto optimal front [19,101,102]. In building research, NSGA-II is often used with simulation tools (e.g., EnergyPlus or TRNSYS) to optimize retrofit solutions, which is often referred to as a simulation-based approach [50,63,72,103,104,105]. However, NSGA-II struggles when the problem has more than three objectives, where maintaining the diversity across the pareto front becomes challenging.

Therefore, to optimize four or more objectives, another type of GA called NSGA-III was developed based on NSGA-II. In such a case, the performance of NSGA-III was compared with NSGA-II for a public building retrofit. NSAG-III outperformed NSGA-II regarding the distribution of non-dominated solutions and it showed significant diversity and convergence compared to NSGA-II [106]. Another variant of NSGA-II, called archive Non-dominated Sorting Genetic Algorithm (aNSGA-II) stores non-dominated solutions, which enhances the convergence towards the Pareto optimal front by maintaining diversity among the solutions [31,44]. Likewise, a preference-based NSGA-III algorithm called prNSGA-III was implemented for scenarios considering present and future climate conditions to optimize four objectives [53]. Although NSGA-III and prNSGA-III exhibit enhanced suitability for high-dimensional objective functions, they involve higher computational complexity. In contrast, aNSGA-II demonstrates dynamic adaptability for enhanced performance, but it has been tested less. Furthermore, the adaptability in handling multiple objectives and the simplicity of conventional GA have attracted some researchers to utilize it in building retrofitting. However, this one struggles with Pareto diversity and depends on predefined weights, which limits the trade-off exploration.

The above-discussed GAs offer major computational savings compared with exhaustive search, highlighting their significance in retrofit optimization. The literature suggests that the choice of a GA variant depends on the complexity of the problem and requires a balance between solution diversity, convergence quality, and computational effort [64,69,107,108].

### 5.3. Simulation-Based Multi-Objective Optimization (SBMOO) Approach

**Figure 8:**
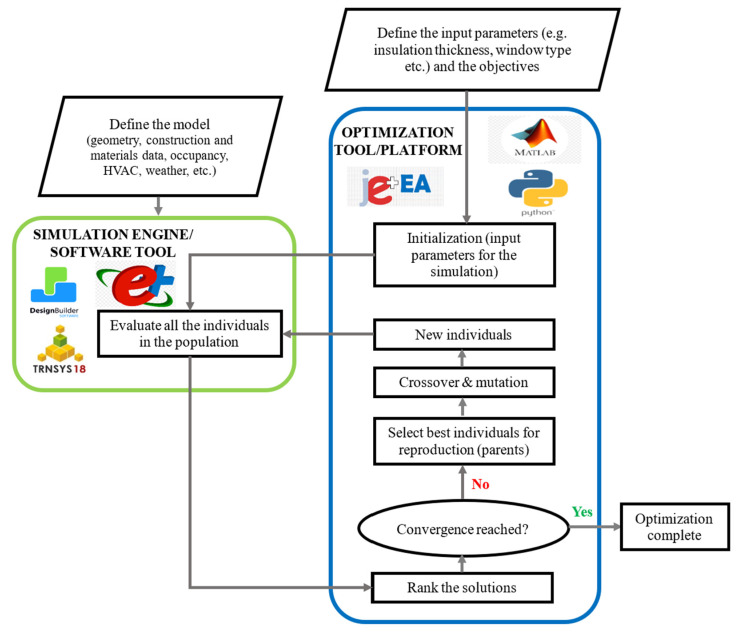
Simulation-based MOO approach.

A simulation-based multi-objective optimization (MOO) integrates an optimization algorithm with energy simulation tools to identify optimal retrofit strategies systematically. This approach balances multiple objectives, such as thermal comfort, energy use, and cost. The step-by-step process of this approach is described below [28,53,72,75]. A visual explanation of the process can be found in Figure 8.

Step 1—Base-building model creation: At the outset, creating a detailed base-building model is critical in the chosen simulation software. This involves defining the building’s layout and geometry, along with general data such as construction materials, thermophysical properties, and the air infiltration rate. Information on occupancy patterns, HVAC system details, and relevant weather data must also be provided.

Step 2—Objective and variable definition: It is necessary to clearly define the objectives that need to be optimized, such as energy consumption, environmental impact, or enhancing occupant comfort. Simultaneously, identify input variables that will be adjusted throughout the optimization process. Performance metrics (output parameters) should also be established to assess how well different strategies achieve the desired objectives.

Step 3—Initial population generation: The optimization process starts with generating an initial population, which consists of various combinations of input parameters (decision variables).

Step 4—Energy performance evaluation: In the next step, the energy simulation tool evaluates each individual (combination of retrofit measures) generated during the initialization phase. This simulation tool calculates the energy performance.

Step 5—Solution ranking: After evaluating all solutions, they are ranked based on their performance across multiple objectives such as energy efficiency, comfort level, and cost-effectiveness.

Step 6—Optimization iteration and genetic operations: In this step, the algorithm observes whether the optimization procedure fulfilled the termination criterion by attaining convergence or reaching the maximum number of generations. If the termination criterion has not been achieved, the proficient solutions are selected as parents for the reproduction. Under genetic operations such as mutation and crossover, the considered parents produce new solutions. This iterative evaluation, classification, selection, crossover, and mutation process persists continuously.

Step 7—Results presentation and Pareto analysis: After optimal solutions are identified in the final stage, the results are presented. These outcomes typically include retrofit strategies that effectively balance multiple objectives. Additionally, for multi-objective optimization problems, the Pareto front is analyzed to understand the trade-offs between competing objectives better. This analysis helps stakeholders make informed decisions by visualizing how different solutions can satisfy varying priorities, guiding the selection of the most suitable retrofit approaches for implementation.

### 5.4. Metamodel-Based Multi-Objective Optimization (MBMOO) Approach

**Figure 9:**
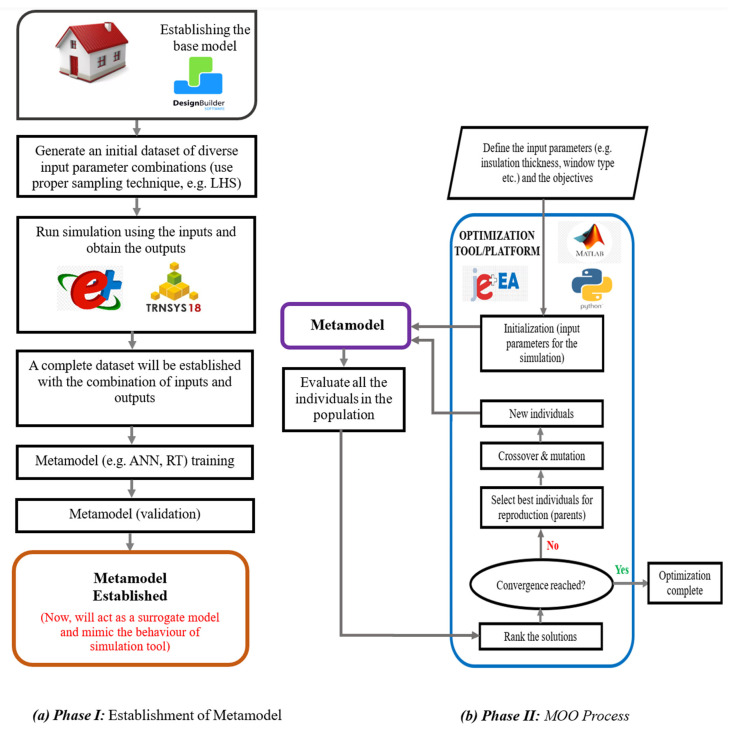
Metamodel-based MOO approach.

Many scholars have noted that simulation-based MOO approaches often face challenges related to time efficiency. The optimization process becomes time-consuming since each evaluation requires running the simulation tool. Metamodel-based optimization has been introduced to mitigate this, where surrogate models or metamodels (e.g., ANN, SVM) mimic the original simulation tool’s performance. A key aspect of this approach is creating a representative database, as these models are trained and validated on it. A common technique for generating compact and representative samples is Latin Hypercube Sampling (LHS). However, determining the sample size remains problem-specific. Scholars recommend beginning with a small sample size to identify biases or inefficiencies. It can be refined over time by progressively increasing the sample size and retraining the model iteratively. Adjustments are made after each iteration to improve performance. The step-by-step process of the metamodel-based simulation framework [17,92,103] is illustrated in the following sections. The graphical illustration is exhibited in Figure 9.

Steps 1 and 2—Defining objectives and developing the base model: The first two steps of the metamodel-based optimization approach closely resemble those of the simulation-based method. The objectives, decision variables (input parameters), and output parameters in these initial stages must be clearly defined. Additionally, using a modeling tool, the base model of the building under study should be developed (refer to Section 5.3 for further details). This foundational step ensures accurate input for the subsequent phases of the optimization process.

Step 3—Implementing sampling techniques for diverse scenarios: An appropriate sampling technique (e.g., LHS) should produce a diverse set of input parameter combinations in this approach. These combinations represent various scenarios of potential retrofit measures, ensuring the solution space is efficiently explored. This diversity is crucial for capturing different retrofit configurations, allowing the surrogate model to simulate different energy-saving strategies while reducing computational costs compared to direct simulation methods.

Step 4—Simulating performance using energy simulation tools: At this stage, various combinations of input parameters are applied to an energy simulation tool (e.g., EnergyPlus, TRNSYS, jEplus) to assess the performance of the building under different retrofit scenarios. The simulation generates outputs for each combination, including energy consumption, comfort levels, environmental impact, etc. This data collection process builds a comprehensive dataset where each row corresponds to a unique combination of input parameters and its respective output metrics, providing a detailed overview of how different measures affect building performance.

Step 5—Training and validating the metamodel: Once the dataset is prepared, the metamodel (e.g., machine learning/regression models) is trained to capture the relationship between the input and output parameters. The training process helps the model learn patterns and correlations within the data. The model is then validated using a separate portion of the dataset (or unseen data) to assess its accuracy in predicting outcomes for new input scenarios. This validation step ensures that the metamodel can reliably predict results for novel combinations of retrofit measures.

Step 6—Integrating the metamodel with optimization algorithm: Once the metamodel is trained, it is integrated into the optimization algorithm (e.g., NSGA-II, NSGA-III, PSO) to serve as a surrogate model for the energy simulation tool. During optimization, the surrogate model rapidly predicts the outputs for novel input combinations, bypassing the need for time-consuming simulations.

Steps 7, 8, and 9—Final optimization and Pareto front analysis: After the above steps, the process continues similarly to steps 5, 6, and 7 in Section 5.3 of the simulation-based approach.

Furthermore, to ensure the accuracy of the optimization results, scholars may perform detailed energy simulations for the optimal solutions provided by the optimization algorithm. This validation step helps confirm that the surrogate model predictions closely align with the real energy simulation outcomes.

Generally, the choice between SBMOO and MBMOO depends on the scale of the problem, accuracy and computational resources. SBMOO is suitable when accuracy is important and evaluations are computationally optimum. Its direct dependency on simulation ensures an accurate representation of complex physical interactions. On the other hand, MBMOO can be a suitable option for computationally expensive problems. By using metamodels, MBMOO enables quick exploration of the solution space. However, the efficiency of the MBMOO approach depends on the sampling strategy, quality of the dataset, metamodel selection, and validation process. An error in the surrogate model can provide misleading retrofit measures. While SBMOO avoids such approximation errors, it suffers from scalability limitations when dealing with complex problems. However, the key comparison between simulation-based MOO and metamodel-based MOO approaches is also shown in tabular form (Table 5) [17,21,31,66,70,109]. Based on the analyzed studies, it also highlights their adoption rates for real-world applications. Due to the robustness and reliance on simulation tools, SBMOO remains the dominant approach (65%). This preference demonstrates the need for accurate outcomes in retrofit decisions. However, the increasing adoption rate of the MBMOO approach (35%) suggests growing interest in computational efficiency when the decision space is complex. This trend is likely driven by advances in machine learning (ML) techniques. As surrogate modeling strategies continue to improve, the MBMOO approach is expected to gain further attraction (Table 5). Moreover, Table 5 also indicates a geographic concentration in the reviewed literature, with more studies from countries such as Italy, Denmark, China, Sweden, Iran, UAE etc. This suggests that evidence from other regions remains limited, which may reduce the broader applicability of some retrofit recommendations.

### 5.5. Simulation and Modeling Tools, MOO Algorithms, and Optimization Platforms Used in Building Retrofitting

**Figure 10:**
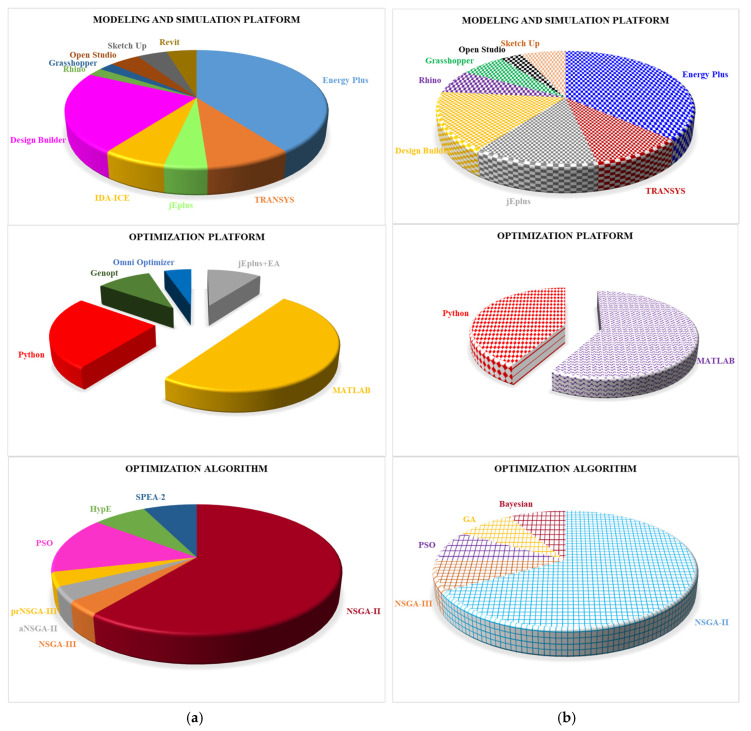
Proportional analysis of platforms and algorithms for SBMOO and MBMOO based on the analyzed studies. (**a**) SBMOO; (**b**) MBMOO.

This section outlines the most commonly used simulation engines, modeling tools, and optimization algorithms in the analyzed SBMOO and MBMOO literature. Figure 10 represents their adoption rates for improved clarity and comparative evaluation visually.

The most implemented energy simulation engine for both SBMOO and MBMOO approaches is EnergyPlus (Figure 10). This open-source dynamic software tool is utilized for energy analysis and thermal load simulation. It gives accurate simulations of building energy performance and allows extensive customization of building systems and schedules for tailored simulations. Additionally, the simulation features of DOE-2 and BLAST, the earlier generation energy modeling, are inherited in EnergyPlus [20,21]. However, EnergyPlus has a limited graphical user interface and requires advanced expertise. To handle this, researchers are keen to use Design Builder (DB) and Open Studio, essential modeling platforms often implemented as the graphical interfaces for EnergyPlus. Due to the user-friendly interface and visualization, the scholars have utilized DB adequately in both SBMOO and MBMOO cases (Figure 10).

Many recent studies [28,53,71,74,104,110] have relied on DB, emphasizing its efficiency in defining architectural geometry, as well as key parameters for the building envelope, internal gains, shading systems, HVAC, and lighting. However, it is computationally intensive and requires licensing. On the contrary, Open Studio also provides a convenient interface for EnergyPlus, while it lacks the capability of advanced energy modeling. In addition, SketchUp has been introduced as a modeling tool to define the geometry of the building [111]. In such a case, energy simulation utilizes EnergyPlus, incorporating building geometry via Open Studio and SketchUp plugins [112]. Moreover, another software tool called Revit is also utilized for the modeling, and later it can be imported to the DB for further analysis and optimization [75,113]. Although Revit acts excellently for early-stage design, it does not have the capabilities of detailed energy simulation.

Besides EnergyPlus, jEplus, TRNSYS, and IDA-ICE are commonly utilized tools for analyzing building energy performance [20,21]. TRNSYS is proficient in modular and renewable energy system modeling but presents complexity and high costs. jEplus enhances parametric simulations with EnergyPlus. However, it lacks robust visualization features. The implementation of jEplus is more prevalent in MBMOO rather than SBMOO, while scholars moderately considered TRANSYS for both cases (Figure 10).

IDA-ICE can accurately model an HVAC system with an intuitive interface, but it has been less used in large-scale optimization due to its limited compatibility. Analysis of the reviewed studies shows that it has only been applied in SBMOO cases (Figure 10). Moreover, Rhino is primarily recognized as a powerful 3D modeling software, while Grasshopper functions as a plugin integrated with Rhino to enable parametric design through visual programming. This combination is widely used in architectural and engineering designs due to its flexibility and precision. Furthermore, Honeybee and Ladybug are extensions of Grasshopper, which serve as interfaces to energy simulation engines like EnergyPlus, facilitating the simulation of environmental factors such as energy performance, daylighting, and climate-based design [41].

An advanced energy simulation and optimization tool like jEplus + EA is designed to work with TRNSYS and EnergyPlus. This is an extended version of the original jEplus tool, a simulation manager that runs batch jobs using energy models with the evolutionary algorithm EA. The jEplus + EA linked NSGA-II is frequently regarded as a significant tool for engineering optimization and architectural design processes. jEplus + EA offers a significant benefit for non-programming designers by eliminating the need to develop intricate optimization algorithms and formulate complex mathematical objective functions [27,92].

MATLAB and Python are the only platforms used in MBMOO cases, while several platforms have been utilized in SBMOO cases. MATLAB is the most widely used platform in both approaches, with Python as the second most prominent (Figure 10). MATLAB has extensive libraries and a global optimization toolbox to support MOO but lacks open-source flexibility. Python also has powerful optimization libraries but has slower execution for complex tasks than MATLAB. Moreover, Genopt has been widely recognized as an optimization tool for building energy performance and system optimization [103]. It integrates well with simulation programs like TRNSYS and EnergyPlus, serving as a platform for multi-objective optimization processes. Some scholars also implemented Omni Optimizer as an optimization platform tool that integrates the principles of Bayesian and GA. This has been utilized to enhance the integrated design of a building, striking a balance between technical and financial factors [40,114]. Furthermore, in both SBMOO and MBMOO approaches, NSGA-II is the predominant optimization algorithm due to its efficiency and robustness in handling MOO. Although it might be computationally intensive, NSGA-III is gaining popularity in MBMOO for handling higher-dimensional objectives. In addition, other types of algorithms, such as aNSGA-II, prNSGA-III, particle swarm optimization (PSO), Strength Pareto Evolutionary Algorithm (SPEA-2), and Hypervolume Estimation (HypE) algorithm, are occasionally used in optimization studies and require attention for further exploration (Figure 10).

### 5.6. Different Machine Learning (ML) Models Used in the Metamodel-Based Approach

In both simulation-based and metamodel-based approaches, GA-based MOO is used to derive optimal solutions for energy performance. Within GA, each individual in a generation represents a distinct set of parameters that are evaluated. These parameters serve as inputs to energy simulations, essential for calculating energy demand, cost, and other factors related to building performance. However, the simulation-based approach necessitates the execution of numerous energy simulations during the optimization process, which can be highly time-consuming and computationally intensive [70]. A surrogate model can approximate the complex energy simulation model to address the challenges of high computational demand and extended processing times, significantly reducing the computational burden [112].

However, constructing the surrogate model requires a dataset that includes input and output variables associated with the evaluation process. So, at the beginning of the metamodel-based approach, a dataset is generated by conducting energy simulations on a limited number of data points that are strategically selected to represent and cover the entire domain [103]. This approach significantly reduces the required energy simulations compared to the simulation-based method, resulting in a much faster MOO process.

In the metamodel-based approach, the generated dataset is divided into training and validation sets to ensure the accuracy and generalizability of the surrogate models. Training involves fitting the ML models to the data, while validation assesses their performance using unseen data. Cross-validation is a common technique used to evaluate model robustness and prevent overfitting. This method splits the data into different training and validation subsets to measure the model’s performance across multiple configurations, minimizing the overfitting risk. However, the accuracy of the surrogate model is often assessed using various statistical indicators, such as coefficient of determination (R^2^), mean absolute error (MAE), root mean square error (RMSE) and mean absolute percentage error (MAPE). These metrics were reported in isolation, as direct comparisons across studies are difficult due to variation in sampling strategy, dataset size and validation procedures. Therefore, it is better to interpret the performance of surrogate models using multiple statistical indicators [115]. Most of the work utilized ML models as the metamodels or surrogate models in their study. ML models such as polynomial regression (PR), multivariate adaptive regression splines (MARS), Gaussian process (GP), support vector machine (SVM), artificial neural network (ANN), regression trees (RT), and multiple linear regression (MLR) are generally used in the context of retrofitting to create the surrogate model [17].

PR is one of the simplest approaches to model the relationships between input and output variables. It is effective for capturing straightforward, linear relationships. However, it may need help with complex, non-linear interactions, which limit its applicability in scenarios requiring more sophisticated modeling. On the other hand, MARS offers a more flexible alternative by modeling non-linear relationships by segmenting the data into multiple linear regions. MARS is adept at capturing complex patterns and interactions in the data, making it suitable for scenarios where the relationship between variables is not purely linear [116]. Additionally, RT can manage non-linear relationships well. Still, there is a risk of overfitting, while MLR is efficient for linear data but struggles with non-linearity and multicollinearity among predictors.

**Figure 11:**
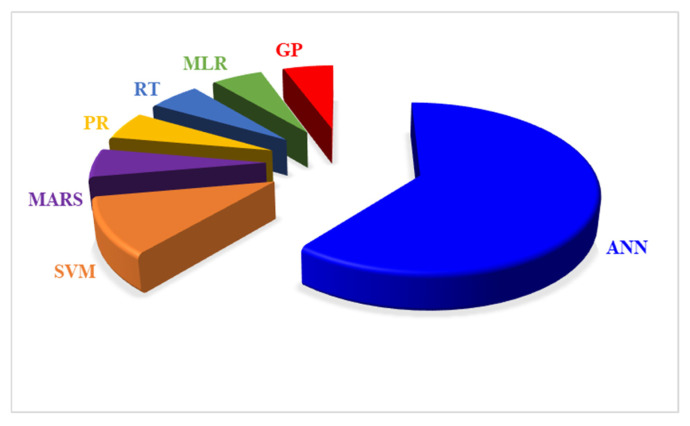
Proportion analysis of surrogate models for MBMOO based on the analyzed literature.

GPs are known for their capability to provide probabilistic predictions and quantify uncertainty. This feature is particularly valuable when understanding the confidence of the model’s predictions is crucial. This characteristic is especially useful in building energy modeling and other areas where understanding the output and associated uncertainty is crucial [117]. SVMs are versatile models that can be applied to regression and classification tasks. They handle high-dimensional spaces effectively and can model non-linear relationships using kernel functions. This makes SVMs a robust choice for complex data sets where interactions between variables are complex [27]. Finally, ANNs are highly flexible and powerful, capable of learning complex patterns and interactions from large datasets. They are the most common surrogate model used in the metamodel-based approach (Figure 11). ANNs can model complex relationships and adapt to a wide range of problems [118]. However, they require significant computational resources and careful tuning to achieve optimal performance [66]. Despite these challenges, their adaptability and strong performance across various optimization problems make them a popular tool in building energy simulations.

Each ML model offers unique advantages and limitations. The choice of model depends on the specific requirements of the surrogate modeling task, such as the need for interpretability, computational efficiency, or the ability to handle non-linearities. Simpler models such as MLR, PR, or RT may be suitable for structured problems with limited complexity, whereas ANN, GP and SVM are appropriate for capturing nonlinear relationships in complex retrofit problems. Therefore, the selection of a surrogate model should be done by doing comparative testing and validation rather than being treated as fixed.

## 6. Synthesized Framework for Retrofit Optimization

**Figure 12:**
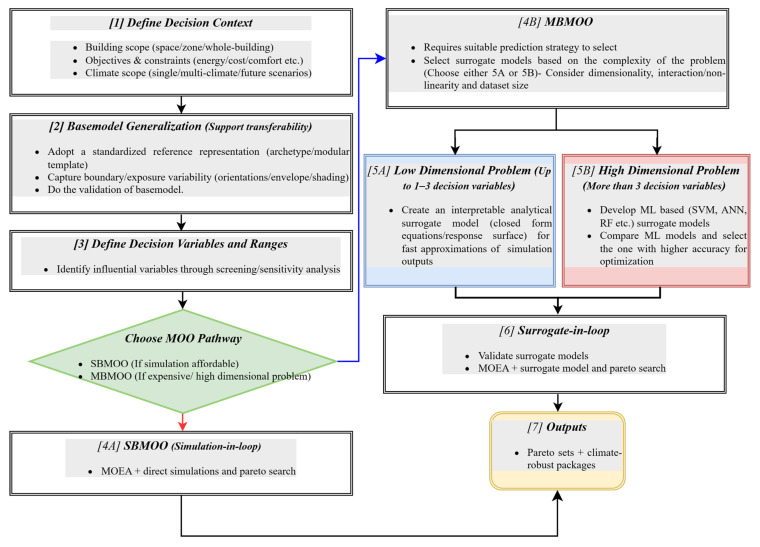
A structured framework for retrofit optimization.

According to the above analyzed SBMOO and MBMOO studies, most remain highly case-specific, limiting their general applicability across different spaces and climates. Table 6 supports these claims and demonstrates that both optimization approaches are commonly utilized for case-specific studies. Multiple climate scopes are associated with simplified base models or archetype, while case studies are linked to single climate scope or did not define the climate condition explicitly. Additionally, envelope parameters such as insulation, infiltration, shading, and glazing strongly influence energy performance. Therefore, evaluating retrofit measures in representative zones can reliably indicate overall building performance [119,120,121]. Nevertheless, while MOO and metamodels are complementary, many existing studies lack standardized prediction frameworks for seamless integration into the MOO process [16,122]. It can be observed from Table 6 that many MBMOO studies adopted fixed surrogate models rather than comparative surrogate selection. Table 6 also depicts that both SBMOO and MBMOO approaches are used for residential building retrofit analysis, whereas the retrofit analysis of non-residential buildings is linked with the MBMOO approach. Therefore, this review presents a framework that outlines a structured pathway for retrofit optimization, addressing two key limitations observed in the literature: case-specific baseline modeling and insufficient justification for surrogate selection in MBMOO studies (Figure 12).

The workflow in Figure 12 establishes the decision context and aims to generalize the baseline model for better transferability across various climates and exposure conditions. The climate scope may be defined using historical weather files for standard assessment. However, if the aim is to analyze long term impacts of retrofit measures, future climatic conditions can be included as the climate scope. Depending on the objectives of the retrofit analysis, the performance metrics may extend beyond cost and energy to include broader life cycle environmental considerations. This consideration can do the balancing between the long-term operational savings and upfront materials impact. Screening/sensitivity analysis identifies key decision variables before selecting the optimization pathway. The problem can follow SBMOO process, if direct simulations are feasible. If the problem involves larger search spaces, multiple interacting decision variables or repeated simulation runs, then the direct simulation may be considered less practical. Otherwise, it utilizes MBMOO, where surrogate selection is guided by the complexity of the problem rather than a fixed model. This is also practically relevant because many real-world retrofits involve only a small number of measures, for which fast and interpretable analytical surrogates may be sufficient, whereas higher-dimensional problems are better suited to ML- based surrogates [124]. The distinction between higher and lower dimensional problems in the framework is provided as a practical guide rather than a fixed threshold, since the applicability of ML or analytical surrogates also depends on variable interactions, complexity of the problem and data availability. Both pathways lead to Pareto-optimal retrofit solutions focused on climate resilience.

Furthermore, the practical relevance of this framework (Figure 12) can also be demonstrated from several recent studies [27,28,70,73,76]. The framework would guide a particular study to the SBMOO branch if the optimization relies on direct simulation outputs and remains feasible for repeated simulations. In studies where a simulation dataset was established and later used to train the surrogate models before optimization, the framework would guide the analysis towards the MBMOO branch for systematic surrogate selection. For studies that included climate change or future climatic conditions, the suggested workflow would encourage the analyst to define the climate scope at the beginning. In this way, the framework would guide the researchers to consider the decision routes in retrofit optimization in a more structured way.

## 7. Conclusions

The reviewed literature demonstrates that climatic conditions strongly influence the choice of retrofit measures. However, most of the studies put emphasis on the implication of passive retrofit measures; effective decision-making requires integrated evaluation of passive, active, and combined retrofit strategies rather than isolated interventions. The review also emphasizes that retrofitting is a multi-objective problem involving trade-offs among energy, cost, thermal comfort, and environmental impact. While existing literature largely focuses on energy and cost, there is a lack of attention to social considerations and life-cycle-based environmental assessments, which hinders the evaluation of long-term sustainability and overall retrofit impacts.

Both SBMOO and MBMOO play important roles in retrofit optimization. Most studies implemented SBMOO approach due to its high-fidelity optimization. However, a growing trend is also observed in the implementation of the MBMOO approach, driven by advances in AI techniques. Although MBMOO improves efficiency through surrogate modeling, it depends heavily on dataset quality, sampling strategy, and model selection. Therefore, the selection between SBMOO and MBMOO should not only consider computational time but also data availability, complexity of the problem and prediction reliability. The review further indicates that many studies are highly case-specific, resulting in limited generalization of models and inadequate justification for surrogate choices in MBMOO. This restricts the applicability of findings across various climates, building types, and retrofit scenarios.

To address these recurring limitations, this review contributed by introducing a synthesized framework for retrofit optimization as a structured decision-support pathway derived from patterns identified in the literature. The review provides a clearer basis for climate responsive retrofit planning and supports context specific retrofit decision, by integrating retrofit strategies, performance indicators, climatic conditions and optimization approaches.

### Future Research Potential

Unlike earlier reviews that depicted retrofit strategies, tools, and optimization methods independently, this review suggests that future work can be structured by linking baseline modeling, climate scope, performance metrics and optimization pathway selection. The following priorities emerge from this perspective.

- Future research should prioritize the development of more transferable retrofit decision pathways. The literature clearly demonstrates that most retrofit studies are case-specific, which limits their applicability across various spaces, geometries, and climates. High-performing models often rely on unique datasets, and the scarcity of data significantly restricts the potential for generalization. Retrofit decisions are predominantly driven by technical optimizations rather than homeowner preferences. Research consistently shows that stakeholders typically prioritize one or two affordable retrofit measures, frequently focusing on insulation upgrades due to financial constraints. This clearly underscores the need for simple, fast, and practical retrofit framework that reflect real-world retrofit practices. Moreover, the literature also indicates that an adaptable base model framework is necessary to quickly pinpoint the energy hot spots where retrofitting is essential.
- A second priority could be the broader integration of environmental and social objectives into retrofit optimization. Although energy and cost are widely considered, other important performance metrics such as LCA, including embodied and operational impact and social factors consisting of occupant behavior, indoor environmental quality, acoustic comfort, and visual comfort remain underexplored.
- There is also a need for comparative studies on SBMOO and MBMOO approaches in terms of practical usefulness, computational efficiency, and accuracy in building retrofits. Developing hybrid frameworks that combine the strengths of SBMOO and MBMOO could be transformative. Moreover, in MBMOO, a transferable prediction workflow is missing which can be served as preliminary optimization screening layer for selecting surrogate models before applying MBMOO. In addition, beyond standard climate zone weather files, future work should also consider future weather scenario for robust climate responsive retrofit studies.

Addressing these directions can support the development of more transferable, efficient, and sustainability oriented retrofit optimization frameworks, improving both the practical usability and climate robustness of future retrofit decision-making.

## Figures and Tables

**Table 1:** Search string formulation for the review.

No	Purpose of the Selection	Aim	Search String
1.	Types of retrofits	Retrofit measures	retrofitting OR “building retrofit*” OR “active strategies” OR “passive strategies” OR renovat* OR “building refurbishment” OR HVAC OR “photovoltaic panels” OR PV AND (retrofit* OR refurbish* OR renovat*)
2.	Energy, environmental, and economic impacts of the retrofit measures.	Impact assessment	assessment OR analysis OR cost OR energy OR LCE OR LCA OR LCC OR “embodied phase” OR “operational phase” OR “demolition phase” OR “climate change” AND (life cycle* OR life-cycle*)
3.	What types of optimizations are used to handle conflicting objectives?	Multi-objective optimization techniques	MOO OR “multi-objective optimi*” OR “genetic algorithm” OR GA OR “NSGA-II” OR “NSGA-III” OR PSO OR “particle swarm optimi*” AND (multi-objective* OR optimi* OR “GA*”)
4.	What prediction strategies are related to building retrofit analysis?	Prediction techniques	“machine learning” OR ML OR “artificial neural network” OR ANN OR “support vector machine*” OR SVM OR “multiple linear regression” OR MLR OR “regression tree*” OR RT OR RF OR “random forest”

Note: * denotes a truncation symbol used in the search string to capture word variants with the same root.

**Table 2:** Different combinations of search strings.

Combinations	No of Papers
(1.2.3.4)	15
(1.2.3.4) + (1.2.3)	87
(1.2.3.4) + (1.2.3) + (1.3.4)	146
(1.2.3.4) + (1.2.3) + (1.3.4) + (1.2.4)	162

‘.’ = AND; ‘+’ = OR.

**Table 3:** Inclusion and exclusion criteria for literature selection.

Criteria Type	Description
Inclusion criteria	Focus on retrofitting existing buildings. Application of multi-objective optimization (SBMOO or MBMOO). Consider climate-specific factors in retrofit strategy selection. Evaluate at least one performance metric (energy, comfort, cost, or environment).
Exclusion criteria	Focus only on the new building design. Theoretical reviews without methodological or optimization insights. Not related to building retrofitting or optimization. AI/ML studies unrelated to building retrofitting or energy performance. No full-text availability.

Note: Filters for publication year (2015–2025), English language, and document type were already applied during the Scopus search.

**Table 4:** Climate responsive retrofit measures and evidence strength from the reviewed studies.

Authors	Climate Type	Common PS	Common AS	Evidence Strength
[5,7,27,28,29,50,51,64,65,66,67,68]	Hot, humid	Wall and roof insulation, glazing/window types, shading, window overhangs, infiltration rate, PCM	Lighting, cooling system upgrades, Heating/cooling setpoint and setback control, PV panel	Strong
[51,64,65,68]	Warm, dry	Wall and roof insulation, window types, overhang specification	Lighting, Heating/cooling setpoint control	Moderate to strong
[5,7,69]	Arid	Wall and roof insulation, PCM	Heating/cooling setpoint control	Moderate
[7,69]	Semi-arid	Wall and roof insulation	Heating/cooling setpoint control	Moderate
[5,29,41,63,64,66,67,68,70]	Cold	Wall and roof insulation, glazing/window types, airtightness, PCM	Heating system upgrades, mechanical ventilation, lighting	Strong
[5,31,43]	Temperate	Wall and roof insulation, glazing, cladding, PCM	Heating pumps upgradation, PV panel	Moderate
[40,71,72,73,74,75]	Climate not explicitly mentioned	Insulation (wall, roof, floor), glazing/window types	Heating/cooling system upgrades, PV panel	Limited for climate-specific conclusions

Note: Evidence strength indicates how consistently similar retrofit measures were reported within each climate group. AS: active strategies, PS: passive strategies.

**Table 5:** Key comparison of SBMOO vs. MBMOO.

Criteria	SBMOO	MBMOO
Computation time	High; typically, hours to days per simulation run.	Significantly lower; after initial training (70–90% reduction in simulation runs).
Dataset requirement	Moderate; runs simulations per scenario directly.	High; requires extensive initial simulation datasets (e.g., LHS sampling).
Typical software tools	EnergyPlus, OpenStudio, Design Builder, Rhinoceros 3D, Grasshopper, Honeybee.	EnergyPlus, jEplus, integrated with ML platforms like MATLAB or Python.
Machine learning model	Not applicable.	ANN, SVM, MLR, Regression Trees (RT).
Optimization algorithms used	GA, NSGA-II, prNSGA-III, aNSGA-II.	Like SBMOO, it is typically integrated with surrogate models.
Common objectives	Energy consumption, thermal comfort, cost optimization, and environmental impact.	Similar objectives (energy, comfort, environment), but easier inclusion of complex metrics can be done.
Accuracy and validation	Accurate outcomes via actual simulation.	A full simulation is needed to validate the results, depending on the dataset quality.
Adaptability for large studies	Less adaptable due to computational constraints.	Suitable for extensive parametric studies.
Application frequency	65% of analyzed studies adopted this approach. Among this, 66.7% targeted residential buildings and 33.3% other types.	35% of reviewed studies used this approach. Of the 35% MBMOO studies, residential buildings accounted for 56.3%, while other types comprised 43.8%.
Example countries	Italy, Canada, Sweden, Denmark, UAE, Jordan, Iran, China, and England.	China, Mexico, England, Switzerland, Portugal, Kuwait, USA, and Argentina.

**Table 6:** Modeling scope and transferability in retrofit optimization studies.

Authors	Building Context	Baseline Model Type	Baseline Generalization	MOO Approach	Metamodel Selection Approach	Climate Scope
[109]	Office	Archetype (space-level)	Limited	MBMOO	Fixed ML model	Multiple
[28]	Residential	Real building (case study)	None	SBMOO	-	Single
[27]	Residential	Real building (case study)	None	MBMOO	ML Model comparison (accuracy-driven)	Single
[70]	Hospital	Archetype (whole-building)	Limited	MBMOO	Fixed ML model	Future Scenario
[123]	Residential	Real building (case study)	None	MBMOO	ML Model comparison (accuracy-driven)	None
[53]	Residential	Real building (case study)	None	SBMOO	-	None
[31]	Residential	Real building (case study)	None	SBMOO	-	Single
[67]	School	Archetype (whole-building)	Limited	SBMOO	-	Multiple
[50]	Residential	Real building (case study)	None	SBMOO	-	Single
[112]	Office	Real building (case study)	None	MBMOO	Fixed ML model	None
[111]	Office	Real building (case study)	None	MBMOO	Regression method	Single
[74]	Residential	Archetype (whole-building)	Moderate	MBMOO	Fixed ML model	None
[41]	Residential	Real building (case study)	None	SBMOO	-	Single
[66]	Residential	Real building (case study)	None	MBMOO	Fixed ML model	Multiple
[75]	University	Real building-space level (case study)	None	SBMOO	-	None
[118]	Residential	Real building (case study)	None	MBMOO	Fixed ML model	Single
[73]	Office	Real building (case study)	None	MBMOO	Fixed ML model	None
[62]	Office	Archetype (space-level)	Limited	SBMOO	-	Multiple
[51]	Residential	Real building (case study)	None	SBMOO	-	Single
[68]	Residential	Archetype (whole-building)	Moderate	MBMOO	Fixed ML model	Multiple
[17]	Residential	Real building (case study)	None	MBMOO	Fixed ML model	None

## Data Availability

No new data were created or analyzed in this study. Data sharing is not applicable to this article.
